# Dance to the rhythm, cautiously: Isolating unique indicators of oscillatory entrainment

**DOI:** 10.1371/journal.pbio.2003534

**Published:** 2017-09-19

**Authors:** Assaf Breska, Leon Y. Deouell

**Affiliations:** 1 Department of Psychology, University of California, Berkeley, California, United States of America; 2 Helen Wills Neuroscience Institute, University of California, Berkeley, California, United States of America; 3 Department of Psychology, Hebrew University, Jerusalem, Israel; 4 Edmond and Lily Safra Center for Brain Sciences, Hebrew University, Jerusalem, Israel

The idea of entrainment of neural oscillations to temporal structure has become a central theory for attentional selection in time [1,2]. In our study [3], we separated unique electroencephalogram (EEG) signatures of oscillatory entrainment to rhythmic streams from those (specifically, inter-trial phase coherence [ITPC]) that overlap with other mechanisms of prediction. Obleser, Henry, and Lakatos’s (OHL’s) comment [4] highlights important issues regarding the study of entrainment mechanisms. We share OHL’s main message: rhythms may be complex and not necessarily isochronous. In fact, finding a stream devoid of any regularity is challenging. This is probably why entrainment mechanisms are functionally important! Yet we should not see entrainment mechanisms as omnipresent, nor should we render them irrefutable. Below, we reflect on some of OHL’s concerns regarding our study.

Oscillatory entrainment predicts increased ITPC for rhythms [2,5], as both our and OHL's models show. However, taking ITPC as evidence for entrainment, without ruling out other explanations, would be making the logical fallacy of "affirming the consequence" (a→b does not imply that b→a). Our study [3] thus asked which electrophysiological phenomena are uniquely associated with entrainment to rhythms instead of with general temporal prediction mechanisms that also operate in rhythmic contexts [6]. We conjectured that if ITPC reflects entrainment it should be sensitive to the level of regularity. However, we found that reducing the level of temporal regularity of streams did not reduce ITPC when predictability was preserved but only did so if predictability was also reduced. OHL's argument that this is explained by predictions-related climbing neuronal activity (CNA; e.g., the contingent negative variation) only recapitulates one of our main arguments [3]. Given this fact, can a repeated-CNA explanation be ruled out from previous ITPC findings, especially in designs with no warning signal? Using near-threshold stimuli [7] reduces onset-responses–driven ITPC but not anticipatory activity.

OHL point out (like we did [3]) that our repeated-interval condition (RIC) was not arrhythmic, explaining observed ITPC levels. Critically though, the RIC was not designed to be arrhythmic but to be less periodic than the isochronous condition (IsoC), which should diminish entrainment signatures. For perceptual judgments evidence (cf. OHL’s demo) that this was achieved, see Fig 1. However, in accordance with OHL's appropriate assertion that rhythmicity is ill defined, we worry that resorting to subjective judgment falls into the same ambiguity of periodicity/predictability that motivated our study. Preferably, the degree to which a sequence is conducive to entrainment of a given oscillation, well defined by frequency and phase, could be objectively quantified. In fact, OHL's model, like ours, predicts a difference in phase (their figure 1b) and a lower ITPC (their figure 1c showing the effect of stimulus onset asynchrony [SOA] variance) in the RIC compared to IsoC. However, being a single-oscillator model with adjustable frequency, OHL’s model cannot attribute the ITPC to specific frequencies. Our multi-oscillator model shows that the expected ITPC is in lower frequencies in the RIC than the IsoC, consistent with the RIC being by design destructive to entrainment at the IsoC frequency. The predictions of both entrainment models are thus inconsistent with the EEG findings of similar ITPC, in the same frequency, and to the same (behaviorally optimal) phase angle in the 2 conditions.

**Fig 1:**
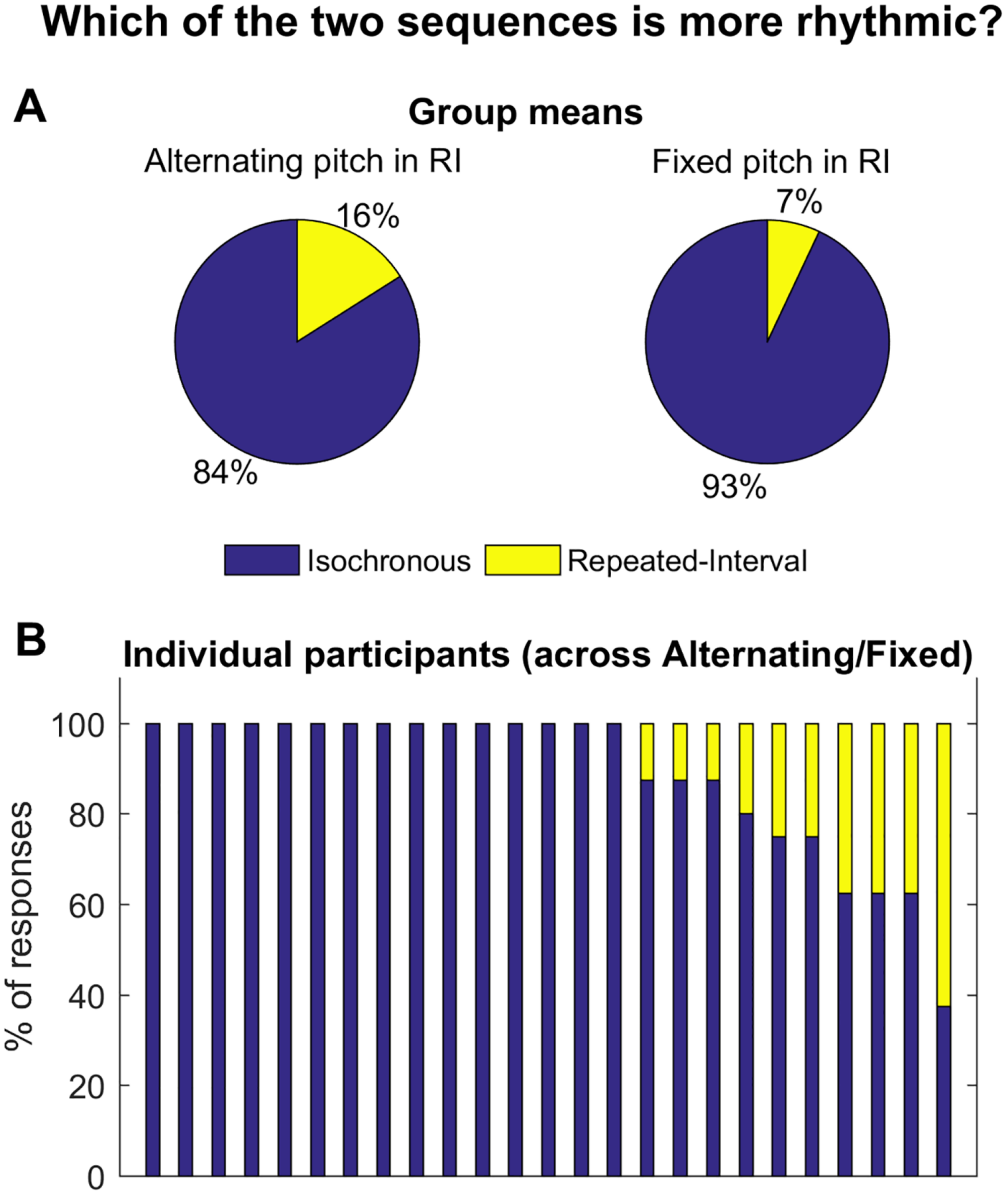
Testing the perceived relative rhythmicity of isochronous and repeated-interval stimulus sequences. Following the suggestion of OHL [4] to test subjective rhythmicity using auditory stimulation, 25 human participants performed a 2-alternative forced-choice online experiment in which they judged which of 2 auditory streams sounded more rhythmic. The procedures of this study adhered to the guidelines of the Declaration of Helsinki and were approved by local Ethics Committee of the Hebrew University of Jerusalem, Israel. (A) Mean distribution of judgments across the group. In each trial (*n* = 8), participants were presented with 2 auditory streams: one isochronous, imitating the sequences of the isochronous condition (IsoC) in [3], and the other non-isochronous, imitating one of the sequences of the repeated-interval condition (RIC) in [3], with different inter-pair jittered intervals in each trial. In each trial, the stimulus onset asynchrony (SOA) of the predictable interval in both sequences were either short (700 ms) or long (1,300 ms). In 4 trials, the stimuli of the RIC condition alternated in pitch, reflecting the alternating colors in [3], while in the other 4 trials the pitch was fixed to prevent the pitch alternation from labeling the RIC condition. The order of conditions (IsoC/RIC) and trial SOA were also counterbalanced, and these 3 factors were orthogonalized. Trials were presented in a random order, different for each participant. After presentation of the 2 sequences, participants indicated which stream sounded more rhythmic using the mouse or keyboard buttons, with no time pressure (see S1 Data for full dataset; see www.soscisurvey.de/abrhythms for exact instructions and full experiment). Participants provided informed consent by agreeing to proceed from the first information screen, and all data were analyzed anonymously. The results show a strong bias towards classifying the IsoC as “more rhythmic” (*t* test comparing percentage of choosing the isochronous as more rhythmic, across all conditions, relative to a null value of 0.5: t_(24)_ = 11.3, *p* = 5 × 10^−11^). (B) Response distributions of individual participants. Twenty-four out of 25 participants chose the IsoC in more than 50% of the trials. Abbreviations: IsoC, isochronous condition; OHL, Obleser, Henry, and Lakatos, authors of the article cited in reference [4]; RIC, repeated interval condition; SOA, stimulus onset asynchrony.

For us, the strongest indication that the 2 sequences, while not differing in ITPC, were not as “rhythmic” (able to entrain) for the brain is the clear behavioral and electrophysiological differences they induced [3]. Crucially, these differences agreed with entrainment theories: the more rhythmic IsoC led to resonance and momentum after stream termination. We emphasize again this overlooked point: our findings support, rather than falsify, entrainment theories. However, they call for proper predictability controls when using ITPC during stream presentation as evidence for entrainment. We join OHL in hoping for clearer definition and careful controls when dissecting predictive mechanisms.

## Supporting information

S1 Data Full dataset of the experiment. (XLSX)

## Abbreviations

CNA: climbing neuronal activity
EEG: electroencephalogram
IsoC: isochronous condition
ITPC: inter-trial phase coherence
OHL: Obleser, Henry, and Lakatos, authors of the article cited in reference [4]
RIC: repeated-interval condition
SOA: stimulus onset asynchrony
